# Ichthyofaunal list of the continental slope of the southern Gulf of Mexico

**DOI:** 10.3897/zookeys.846.31944

**Published:** 2019-05-16

**Authors:** José Martín Ramírez, Ana Rosa Vázquez-Bader, Adolfo Gracia

**Affiliations:** 1 Posgrado en Ciencias del Mar y Limnología, Universidad Nacional Autónoma de México Universidad Nacional Autónoma de México México Mexico; 2 Posdoc. Unidad Académica de Ecología y Biodiversidad Acuática, Instituto de Ciencias del Mar y Limnología, Universidad Nacional Autónoma de México Universidad Nacional Autónoma de México México Mexico; 3 Unidad Académica de Ecología y Biodiversidad Acuática, Instituto de Ciencias del Mar y Limnología, Universidad Nacional Autónoma de México, A.P.70-305, Ciudad de México, 04510, México Universidad Nacional Autónoma de México México Mexico

**Keywords:** Deep water, fishes, new records, species richness

## Abstract

Four oceanographic cruises were carried out between April 2011 and May 2013 on the continental slope of the southern Gulf of Mexico (GoM) in a depth range of 290 to 1200 m on board the R/V JUSTO SIERRA. A total of 91 trawls covered a total swept area of 170.49 hectares. We recorded 177 fish species belonging to 80 families. Fifteen species extended their distribution into the south of the gulf and 37 increased their depth ranges. Five species could have commercial importance: *Aphanopuscarbo* Lowe, 1839; *Hydrolagusmirabilis* (Collett, 1904); *Helicolenusdactylopterus* (Delaroche, 1809); *Lophiusgastrophysus* Miranda Ribeiro, 1915, and *Merlucciusalbidus* (Mitchill, 1818). The most abundant species were *Polymixialowei* Günther, 1859; *Parasudistruculenta* (Goode & Bean, 1896); *M.albidus*, *Chlorophthalmusagassizi* Bonaparte, 1840; *Dibranchusatlanticus* Peters, 1876; *Nezumiaaequalis* (Günther, 1878); *Yarrellablackfordi* Goode & Bean, 1896; and *Laemonemabarbatulum* Goode & Bean, 1883. High values of fish species richness, diversity, and evenness were registered throughout the study area. A high percentage of the fish species (97%) collected during this project are distributed in the entire GoM. Most of the species showed a wide depth distribution; however, a vertical zonation of species can be observed.

## Introduction

The Gulf of Mexico (GoM) is one of the most productive and economically important ecosystems in the world (Cato 2009, Tunnell 2009), and its large biodiversity makes it one of the most diverse seawater bodies (Felder et al. 2009). Due to its ecological and economic importance, ichthyofauna studies initially focused on commercial species. Research on fish biodiversity in the GoM, which began in 1850 (Darnell and Defenbaugh 1990), became more systematic and extensive since 1950. A total of 1541 species has been reported in the GoM in the different types of habitats that exist in this large ecosystem (McEachran 2009). Nevertheless, more emphasis has been placed on coastal regions because they are more accessible and economical to survey compared to deeper areas and the open sea.

Few investigations about fish biodiversity have been conducted on the continental slope, and most have focused on different ecological aspects of demersal fish communities in the northern part of the GoM (Pequegnat et al. 1990, Powell et al. 2003). More than 126 mesopelagic fish species were found in this region by Ross et al. (2010), who compared the composition of mesopelagic fishes in three different habitats located at depths between surface and 1000 m. Sulak et al. (2007) documented 53 benthic fishes associated with deep water coral habitats in the north-central gulf. McEachran and Fechhelm (1998) produced one of the most complete ichthyological inventories for the GoM and for the Caribbean Sea’s continental slope. In addition, Anderson et al. (1985), Saavedra-Díaz et al. (2004) and Paramo et al. (2015) issued complementary reports of 44 species in this region. Others studies of the deep-water fishes in the Caribbean, but concerning to deep reef fishes have been conducted by Colin (1974); Thresher and Colin (1986); Baldwin and Robertson (2014); Baldwin et al. (2016) and Quattrini et al. (2017).

Since there were not studies of fish communities in the southern deep-water of the GoM, the ichthyological inventory of this ecosystem is not yet completed. The Mexican portion of the GoM deep water has recently become an area of interest because of its oil extraction potential (PEMEX 2016) and its potential fishing resources, where at least three important shrimp species have recently been discovered (Gracia et al. 2010). In a potential scenario of exploitation of both living and non-living resources of deep waters of the GoM, it is crucial to acquire more knowledge about this ecosystem. Biodiversity inventories need to be developed to understand, manage, and conserve these resources.

In this study we present information of the fish biodiversity of the scarce explored continental slope of the southern GoM. Our study is the first one that systematically analyzes the deep-water fish fauna in this region.

## Materials and methods

**Figure 1.**
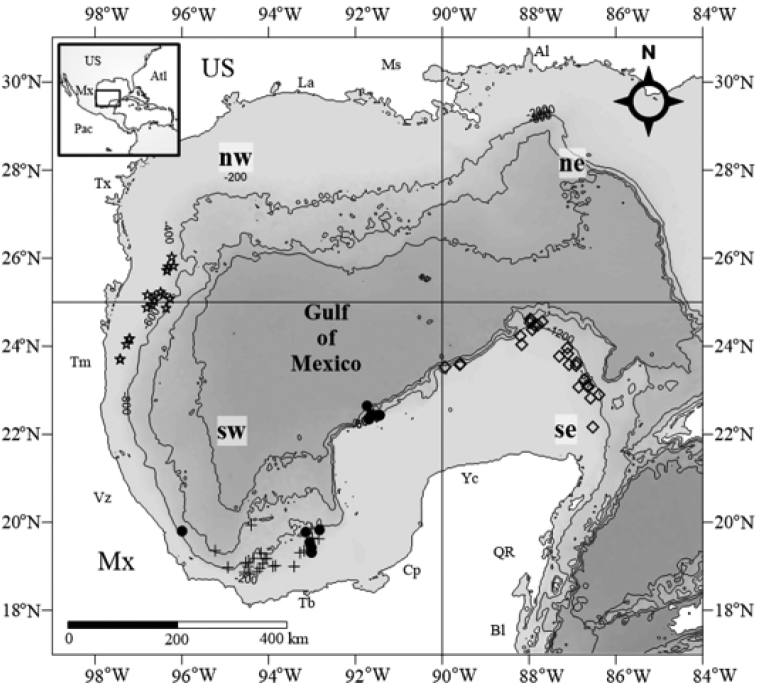
Locations of the oceanographic cruises: COBERPES 2; COBERPES 3; COBERPES 4; and COBERPES 5. Abbreviations: ne: north-east; nw: north-west; se: south-east; sw: south-west; Al: Alabama; Atl: Atlantic; Bl: Belize; Cp: Campeche; La: Louisiana; Ms: Mississippi; Mx: Mexico; Pac: Pacific; QR: Quintana Roo; Tb: Tabasco; Tm: Tamaulipas; Tx: Texas; US: United States; Vz: Veracruz; Yc: Yucatan. Gulf of Mexico division taken from Felder et al. (2009).

The GoM is in a subtropical region that measures 1600 km from east-to-west and 1300 km from north-to-south. It is influenced by the Caribbean Sea due to the transport of water masses via the Loop Current flowing into the gulf through the Yucatan Channel and out of the gulf through the Straits of Florida. Winds, especially in winter also impact gulf circulation (Monreal-Gómez et al. 2004) (Fig. 1).

This research forms part of the project “Biodiversity and Potential Fishing Resources in Deep waters of the Gulf of Mexico," through which oceanographic cruises (Benthic communities and potential fishing resources in the Gulf of Mexico deep waters, COBERPES) were conducted on the Mexican continental slope of the GoM on board the R/VJUSTO SIERRA of the Universidad Nacional Autónoma de México.

Four oceanographic cruises were carried out from April 2011 to May 2013: COBERPES 2 and COBERPES 3 on the Yucatan Slope; COBERPES 4, off the coast of Tamaulipas and COBERPES 5 on the Campeche Bank (Table 1). The benthic megafauna of soft bottom substrates was sampled in a depth range of 290–1200 m, using a semi-commercial shrimp trawling net with an 18m mouth, a 4.5cm mesh and a 1.5cm cod-end opening. Since information about sea bottom was lacking, sea bed was previously explored using a Multihaz EM 300 echo sounder and a Topas PS-18 sub-bottom profiler. After finding adequate substrate, 30-minute trawls were performed at an average velocity of 77.16 m/min. The distance of each tow was determined by GPS readings. Fauna samples were sorted by taxonomic groups, weighed, and preserved in 10% formalin on board.

**Table 1.** Geographic location and data on oceanographic cruises.

Cruise	Date	Geographic locations				Number of samples	Area (ha)
**COBERPES 2**	07–15 Apr 2011	23°02'46"N, 86°26'34"W	23°30'98"N, 89°49'42"W	24°22'60"N, 87°42'98"W	22°53'05"N, 86°15'49"W	28	46.79
**COBERPES 3**	13–19 Nov 2011	22°25'65"N, 91°26'49"W	19°19'38"N, 93°02'54"W	22°23'93"N, 91°37'41"W	19°33'82"N, 93°01'46"W	20	34.87
**COBERPES 4**	23–30 Aug 2012	23°30'73"N, 97°12'79"W	25°47'30"N, 96°14'83"W	24°54'93"N, 96°36'91"W	25°45'97"N, 96°13'05"W	20	38.58
**COBERPES 5**	22–31 May 2013	19°03'92"N, 94°05'53"W	18°45'66"N, 94°22'13"W	19°00'80"N, 93°50'35"W	19°48'22"N, 92°59'11"W	23	50.25

In the laboratory, fishes were identified to species level. The names, authorities, and years of the descriptions were cross-referenced against the Eschmeyer database (2017), as well as the geographic and bathymetric distribution of the species was consulted in different web sites: Ocean Biogeographic Information System (OBIS 2018); Smithsonian National Museum of Natural History; Biodiversity of the Gulf of Mexico Database (Moretzsohn et al. 2017); Texas A & M University Corpus Christi, Harte Research Institute for Gulf of Mexico Studies (2017); FishNet 2 (2013); World Register of Marine Species (WoRMS 2017) and FishBase (Froese and Pauly 2017). Each individual was measured, weighed, preserved in 70% alcohol, and retained in the Reference Collection of the Laboratorio de Ecología Pesquera de Crustáceos del Instituto de Ciencias del Mar y Limnología, UNAM. Number of fish species vs. sampling effort was analyzed to determine sample size using the Clench model (Jiménez-Valverde and Hortal 2003) and the freeware Stimates v8 (Colwell 2006). With the biological data was examined the abundance (individuals/ha), richness (number of species), diversity (Shannon and Wiener 1963), and evenness (Pielou 1977) of the fish communities in different sampling areas. The bathymetric distribution of the species was recorded considering the average depth of each trawl.

## Results

**Figure 2.**
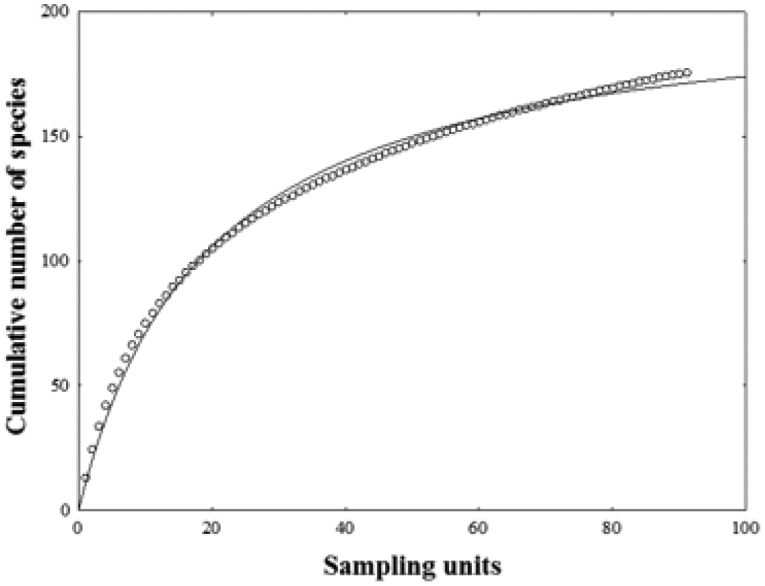
Plot for fish species accumulation for the total sample. Key: circles, random curve; continuous line, fit curve of Clench function (*Sn* = (10.79*n)/(1+(0.0520*n). Each sample unit consisted of 30 minutes trawl at an average speed of 77.16 m/min (2.5 knots).

Ninety-one trawls covering a 290–1200 m depth range were done in the different regions of the southern GoM during the four oceanographic cruises. The numbers of successful trawls at each depth stratum were 300 m: 17; 400 m: 11; 500 m: 16; 600 m: 8; 700 m: 11; 800 m: 11; 900 m: 6; and 1000 m: 4, corresponding to 170 hectares total swept area. Seven trawls failed (Table 1). A total of 9781 fishes was caught, belonging to 80 families and 177 species (Table 2). The species accumulation curve related to the number of samples did not reach a clear asymptote; however, data adjusted with a Clench model suggests that 91% species richness of the southern GoM continental slope was recorded (Fig. 2).

**Table 2.** List of the fish community. Presence and depth distribution ranges of fish species in the different cruises and literature reported (McEachran 2009, Ocean Biogeographic Information System (OBIS), Eschmeyer 2017, Moretzsohn et al. 2017, FishNet 2 2013, World Register of Marine Species 2017, and Froese and Pauly 2017). Key: * species which extended their distribution into the south of the Gulf of Mexico; ne: north-east; nw: north-west; se: south-east; sw: south-west; Al: Alabama; Atl: Atlantic; Bh: Bahamas; Bl: Belize; Cp: Campeche; Cb: Caribbean; Cu: Cuba; La: Louisiana; Ms: Mississippi; Mx: Mexico; QR: Quintana Roo; Tb: Tabasco; Tx: Texas; Tm: Tamaulipas; US: United States; Vz: Veracruz; Yc: Yucatan. ** Species which extended their depth ranges.

	COBERPES cruises					Reported distribution and depth range (m)
Specie	2	3	4	5	Species depth range (m)	
*Antigoniacapros* Lowe, 1843		X			296	entire/50–900
*Antigoniacombatia* Berry & Rathjen, 1959	X				308	Fl, Al, Bl/68–585
*Aphanopuscarbo* Lowe, 1839			X		823	Atl, Vz/200–2300
*Apristuruslaurussonii* (Saemundsson, 1922)	X				562–937	Ms, Al, Tx, Fl, Mx/500–1000
*Argentinageorgei* Cohen & Atsaides, 1969**	X	X	X	X	300–825	entire/220–457
*Argyropelecusaculeatus* Valenciennes, 1850	X		X	X	436–825	entire/100–2056
*Aristostomiastittmanni* Welsh, 1923	X				974	entire/100–2000
*Astronesthessimilus* Parr, 1927				X	611	entire/0–800
*Atractodenchelysphrix* Robins & Robins, 1970**	X			X	534–600	Cb, Fl, Cu, Vz/385–425
*Baldwinellaaureorubens* (Longley, 1935)		X		X	300–611	Mx/91–610
*Baldwinellavivanus* (Jordan & Swain, 1885)	X		X	X	300	Mx/20–610
*Barathronusbicolor* Goode & Bean, 1886					846	entire/366–1561
*Barbantuscurvifrons* (Roule & Angel, 1931)			X		953	ne, nw, Fl/0–4500
*Bathyclupeaargentea* Goode & Bean, 1896**	X		X	X	300–677	entire/366–677
*Bathycongrusdubius* (Breder, 1927)				X	327	entire/120–886
*Bathycongrusvicinalis* (Garman, 1899)	X				477	Mx, US, Cb/101–503
*Bathygadusfavosus* Goode & Bean, 1886	X	X			904–1068	entire/770–2745
*Bathygadusmacrops* Goode & Bean, 1885**	X	X	X	X	494–1068	entire/200–777
*Bathygadusmelanobranchus* Vaillant, 1888	X	X	X	X	602–1071	entire/400–2600
*Bathypteroisbigelowi* Mead, 1958	X		X		534–780	entire/377–986
*Bathypteroisgrallator* (Goode & Bean, 1886)		X			953	entire/878–4720
*Bathypteroisquadrifilis* Günther, 1878	X				865	entire/462–1408
*Bathypteroisviridensis* (Roule, 1916)	X		X	X	593–904	entire/476–1477
*Bathyurocongervicinus* (Vaillant, 1888)	X	X			477	ne, nw, Tm/100>1000
*Bembropsanatirostris* Ginsburg, 1955**	X	X	X	X	300–611	entire/82–538
*Bembropsgobioides* (Goode, 1880)**	X	X	X	X	300–825	entire/82–740
*Benthodesmussimonyi* (Steindachner, 1891)*	X			X	436–500	ne/200–900
*Benthodesmustenuis* (Günther, 1877)	X	X	X	X	300–825	nw, ne, Mx/200–850
*Bolinichthyssupralateralis* (Parr, 1928)	X		X	X	599–677	entire/40–850
*Bregmacerosatlanticus* Goode & Bean, 1886				X	300–462	entire/50–2000
*Bregmaceroscantori* Milliken & Houde, 1984***	X				812	ne/0–475
*Bregmaceroshoudei* Saksena & Richards, 1986***	X		X		346–611	ne/>50
*Brotulotaenianigra* Parr, 1933***			X	X	800–953	Atl/1000–1100
*Caulolatiluscyanops* Poey, 1866		X			300–500	entire/45–459
*Chascanopsettalugubris* Alcock, 1894	X				358–426	entire/60–3210
*Chauliodussloani* Bloch & Schneider, 1801	X	X	X	X	300–953	entire/0–4700
*Chaunaxpictus* Lowe, 1846	X	X	X	X	321–865	ne, nw, Tb/200–978
*Chiasmodon* sp.				X	780	ne, Tb, QR
*Chlorophthalmusagassizi* Bonaparte, 1840	X	X	X	X	300–825	entire/50–3000
*Citharichthysdinoceros* Goode & Bean, 1886	X				336–423	ne, QR, Bl, Cu/180–2000
*Coccorellaatlantica* (Parr, 1928)			X		995	entire/50–1000
*Coelorinchuscaribbaeus* (Goode & Bean, 1885)**		X	X	X	300–825	entire/200–700
*Coelorinchuscaelorhincus* (Risso, 1810)	X		X	X	436–800	entire/90–1485
*Coelorinchusocca* (Goode & Bean, 1885)	X	X		X	321–820	entire/400–2220
*Coelorinchusventrilux* Marshall & Iwamoto, 1973	X	X	X	X	300–534	se, sw/300>500
*Colocongermeadi* Kanazawa, 1957		X	X		494–846	Tm, Vz, ne, nw/366–925
*Conocaramacropterum* (Vaillant, 1888)**	X	X		X	354–1071	Mx/800–2200
*Coryphaenoidesalateralis* Marsahll & Iwamoto, 1973	X				904	Mx/1035–1116
*Coryphaenoidesmexicanus* (Parr, 1946)	X				534–937	Mx/110–1600
*Coryphaenoideszaniophorus* (Vaillant, 1888)	X		X	X	677–1065	entire/400–2775
*Crurirajarugosa* Bigelow & Schroeder, 1958	X	X	X		321–825	Mx/366–1007
*Cyttopsisrosea* (Lowe, 1843)	X	X	X	X	300–825	Mx/100>1000
*Dactylobatusclarkii* (Bigelow & Schroeder, 1958)	X				626	Mx/366–1000
*Diaphusdumerilii* (Bleeker, 1856)	X		X		423–823	entire/0–850
*Diaphusfragilis* (Tåning, 1928)			X		823	entire/15–1313
*Dibranchusatlanticus* Peters, 1876	X	X	X	X	300–1071	entire/22–1300
*Dicroleneintroniger* Goode & Bean, 1883		X	X	X	321–1071	entire/200–1785
*Diplacanthopomabrachysoma* Günther, 1887	X		X		494–766	entire/439–1670
*Dipturusoregoni* (Bigelow & Schroeder, 1958)		X			611	Mx/369–1079
*Dipturusteevani* (Bigelow & Schroeder, 1951)				X	540–800	Cp/311–940
*Diretmoidespauciradiatus* (Woods, 1973)**	X	X	X	X	321–800	entire/0–600
*Epigonusdenticulatus* Dieuzeide, 1950	X	X		X	354–800	Mx/130–830
*Epigonusmacrops* (Brauer, 1906)			X		766–823	entire/550–1300
*Epigonusoccidentalis* Goode & Bean, 1896	X			X	573–700	Vz, Tm/360–737
*Epigonusoligolepis* Mayer, 1974	X				540–619	Mx/380–660
*Epigonuspandionis* (Goode & Bean, 1881)		X	X	X	419–494	Cp/200–600
*Epigonuspectinifer* Mayer, 1974			X		346–677	Mx/100–1200
*Eptatretuscaribbeaus* Fernholm, 1982***	X				597	Cb/300–400
*Espringeriafolirostris* Bigelow & Schroeder, 1951		X	X	X	354–800	ne, nw, se, sw/50–1052
*Etmopterusschultzi* Bigelow, Schroeder & Springer, 1953	X	X	X	X	300–852	entire/200–1000
*Etmopterusvirens* Bigelow, Schroeder & Springer, 1953	X	X	X	X	392–800	Mx/100–1000
*Gadellaimberbis* (Vaillant, 1888)	X	X	X	X	300–974	Mx, Cb, Cu/70>900
*Gadomusarcuatus* (Goode & Bean, 1886)**	X	X	X	X	321–1068	entire/610–1370
*Gadomusdispar* (Vaillant, 1888)		X	X		611–677	Tm/548–1105
*Gadomuslongifilis* (Goode & Bean, 1885)**	X	X	X	X	321–1071	entire/630–2168
*Galeusarae* (Nichols, 1927)	X	X			358–780	Mx/250–750
*Gibberichthyspumilus* Parr, 1933	X				746	entire/300–1100
*Giganturachuni* Brauer, 1901	X				540	entire/0–1830
*Gymothoraxkolpos* Böhlke & Böhlke, 1980	X				336	entire/30–300
*Halieutichthysaculeatus* (Mitchill, 1818)		X			611	entire/8–820
*Halosaurusovenii* Johnson, 1864	X	X	X	X	321–1068	entire/300>2000
*Helicolenusdactylopterus* (Delaroche, 1809)	X				426	Mx/50–1100
*Hemantiasleptus* (Ginsburg, 1952)		X			611	entire/35–640
*Heptranchiasperlo* (Bonnaterre, 1788)				X	436	entire/0–1000
*Hollardiahollardi* Poey, 1861	X	X		X	300–800	Mx/230–915
*Hoplostethusmediterraneus* Cuvier, 1829*	X		X	X	354–800	ne/100–1750
*Hoplunnistenuis* Ginsburg, 1951**		X		X	302–611	entire/30>400
*Hydrolagusalberti* Bigelow & Schroeder, 1951		X	X	X	494–1068	entire/348–1470
*Hydrolagusmirabilis* (Collett, 1904)	X		X	X	462–812	entire/450–1933
*Hygophumreinhardtii* (Lütken, 1892)	X				611	entire/0–1100
*Hymenocephalusaterrimus* Gilbert, 1905		X			354–540	entire/340–1348
*Hymenocephalusbillsam* Marshall & Iwamoto, 1973	X				573–711	entire/400–900
*Hymenocephalusitalicus* Giglioli, 1884	X	X	X	X	428–800	entire/100–1400
*Ijimaiaantillarum* Howell Rivero, 1935**	X	X	X	X	462–1068	entrire/439>700
*Laemonemabarbatulum* Goode & Bean, 1883*	X	X	X	X	426–937	QR/50–1620
*Leptodermamacrops* Vaillant,1886	X	X	X	X	700–1065	Mx/500–2000
*Leucorajagarmani* (Whitley, 1939)**	X	X	X	X	300–800	Mx/37–530
*Leucorajalentiginosa* (Bigelow & Schroeder, 1951)**	X	X	X	X	346–852	entire/53–538
*Lophiodesberoe* Caruso, 1981*	X	X		X	462–735	ne/347–860
*Lophiodesmonodi* (Le Danois , 1971)**	X	X		X	419–800	ne, se/366–549
*Lophiodesreticulatus* Caruso & Suttkus, 1979	X				590–619	entire/64–820
*Lophiusgastrophysus* Miranda Ribeiro,1915	X				599	entire/40–700
*Luciobrotulacorethromycter* Cohen,1964	X	X			606–846	Mx/220–1830
*Macroramphosusscolopax* (Linnaeus, 1758)*	X				308	ne, nw, Cu/25–600
*Malacocephaluslaevis* (Lowe, 1843)	X	X	X	X	300–800	entire/200–1000
*Malacocephalusoccidentalis* Goode & Bean, 1885	X	X	X	X	308–800	entire/140–1495
*Merlucciusalbidus* (Mitchill, 1818)	X	X	X	X	300–852	entire/80–1170
*Monolenesessilicauda* Goode, 1880	X	X			336–1046	ne, nw, sw/0>3000
*Monomitopusagassizii* (Goode & Bean, 1896)	X	X	X	X	300–1071	entire/48–1125
*Myctophumnitidulum* Garman,1899			X		823	entire/0–1537
*Nemichthysscolopaceus* Richardson, 1848		X			321	ne, nw, Yc, Cu/100–4337
*Neoepinnulaamericana* (Grey, 1953)			X	X	300–370	Yc/0–600
*Neoscopelusmacrolepidotus* Johnson, 1863*	X	X	X	X	300–852	ne, nw, Cu, Tb/300–1180
*Neoscopelusmicrochir* Matsubara, 1943*	X			X	300–814	ne, nw, Cu, Bh/60>900
*Nettastomamelanurum* Rafinesque, 1810	X	X	X	X	300–852	entire/37–1647
*Nezumiaaequalis* (Günther, 1878)	X	X	X	X	321–973	entire/200–2320
*Nezumiacyrano* Marshall & Iwamoto, 1973**	X	X	X	X	321–1071	entire/400–1324
*Nezumiasuilla* Marsahll & Iwamoto, 1973	X				904–1046	entire/860–1500
*Oxinotuscaribbaeus* Cervigón, 1961**				X	800	Yc/402–457
*Parasudistruculenta* (Goode & Bean, 1896)	X	X	X	X	300–846	entire/0>1000
*Parazenpacificus* Kamohara, 1935	X				300	Cp, Cu/145–512
*Peristedionecuadorense* Teague, 1961*	X	X			392–814	ne, nw/324–910
*Peristediongreyae* Miller, 1967**	X	X	X	X	300–1071	entire/60–914
*Peristedionlongispatha* Goode & Bean, 1886	X			X	302–553	entire/101–787
*Peristedionminiatum* Goode, 1880		X	X	X	300–500	entire/64–910
*Peristedionthompsoni* Fowler, 1952*	X				358–423	ne, nw, Cu/115–475
*Peristediontruncatum* (Günther, 1880)	X	X	X		336–852	Vz, Yc/155–910
*Photostomiasguernei* Collett, 1889	X				540–772	entire/500–3100
*Poecilopsettabeanii* (Goode, 1881)	X	X	X	X	300–825	entire/155–1636
*Polyipnusasteroides* Schultz, 1938*	X			X	300–820	ne, nw/0>1000
*Polymetmethaeocoryla* Parin & Borodulina, 1990	X	X	X	X	300–953	entire/165–1400
*Polymixialowei* Günther, 1859	X	X	X	X	300–825	entire/0>2000
*Pontinuslongispinis* Goode & Bean, 1896**	X	X		X	300–611	entire/50–440
*Prionotusalatus*Goode&Bean,1883**		X			611	Yc/35–457
*Prionotusstearnsi* Jordan & Swain, 1885	X	X	X		308–346	entire/11–549
*Pristipomoidesmacrophthalmus* (Müller & Jelks, 1848)**		X			611	ne, nw, Cp/110–550
*Promethichthysprometheus* (Cuvier, 1832)	X			X	540–609	ne, Cu, Yc/80–800
*Pseudomyrophisfrio* (Jordan & Davis, 1891)**		X			494	sw, Atl, Yc/0–180
*Pseudorajafischeri* Bigelow & Schroeder, 1954	X				534–580	Yc/412–576
*Rinoctesnasutus* (Koefoed, 1927)		X			1068	ne, nw, Yc/1000–4337
*Rouleiniamaderensis* Maul, 1948	X	X	X		852–1068	ne, Cu/595–1450
*Sauridacaribbaea* Breder, 1927	X		X		308–422	entire/4–460
*Sauridanormani* Longley, 1935	X	X		X	300–611	entire/25–550
*Scopelosaurussmithii* Bean, 1925			X		953	ne, Vz/50>3000
*Scorpaenadispar* Longley & Hildebrand, 1940**	X	X	X	X	300–812	entire/0>500
*Scyliorhinusretifer* (Garman, 1881)	X			X	300–812	entire/36–750
*Setarchesguentheri* Johnson, 1862	X				392	ne, nw, Yc, QR/150–780
*Sigmopselongatum* (Günther, 1878)	X	X	X	X	494–1068	entire/25–1463
*Sphagemacrurusgrenadae* (Parr, 1946)**	X	X	X	X	820–1071	entire/1000–1960
*Squalogadusmodificatus* Gilbert&Hubbs,1916	X		X		865–995	entire/50–1740
*Squaluscubensis* Howell Rivero, 1936**	X	X	X	X	300–608	entire/60>500
*Squatinadumeril* Lesueur, 1818			X		354–370	entire/0–1375
*Steindachneriaargentea* Goode & Bean, 1886			X	X	300–370	entire/350–550
*Stephanoberyxmonae* Gill, 1883	X	X	X		628–953	entire/945–4777
*Sternoptyxdiaphana* Hermann, 1781	X	X	X		577–1065	entire/300–3676
*Sternoptyxpseudobscura* Baird, 1971	X		X		628–953	entire/0>3000
*Stomiasaffinis* Günther, 1887	X	X			611–772	entire/0–3180
*Symbolophorusrufinus* (Tåning, 1928)				X	327	entire/0–3000
*Synagropsbellus* (Goode & Bean, 1896)	X	X	X	X	300–974	entire/00>900
*Synagropsspinosus* Schultz, 1940**	X	X	X	X	300–825	entire/0–544
*Synaphobranchusaffinis* Günther, 1877					820	entire/290–2400
*Synaphobranchusoregoni* Castle, 1960	X	X	X	X	377–1071	entire/45–1900
*Synchiropusagassizii* (Goode & Bean, 1888)	X	X			336–426	Mx, Cb, Cp/0–500
*Tetronarcenobiliana* (Bonaparte, 1835)				X	540	ne, nw, Cp, Yc/0–530
*Thaumatichthysbinghami* Parr, 1927**	X				820	ne, nw, Cb/1100–4032
*Trachonurussulcatus* (Goode & Bean, 1885)**	X	X	X	X	626–1068	entire/700–1500
*Trachyscorpiacristulata* (Goode & Bean, 1896)					619–628	ne, Cb, Mx/130–1100
*Urophyciscirrata* (Goode & Bean, 1896)**		X	X	X	300–825	entire/27>700
*Veneficaprocera* (Goode & Bean, 1883)	X		X	X	327–953	ne, nw, Tm, Vz/326–2340
*Ventrifossamacropogon* Marshall, 1973**	X	X	X		300–846	Tm, Yc/439–1000
*Ventrifossamucocephalus* Marshall, 1973***	X	X	X	X	300–814	ne, Cb/450–732
*Xenocephalusegregius* (Jordan & Thompson, 1905)	X		X		370–423	entire/180–440
*Xenodermichthyscopei* (Gill, 1884)	X			X	590–865	ne, nw, Vz, Tb/100–2650
*Xenolepidichthysdalgleishi* Gilchrist, 1922	X		X		346–547	Tm, Cp/90–900
*Yarrellablackfordi* Goode & Bean, 1896	X	X	X	X	321–1071	entire/350–1000
*Zalieutesmcgintyi* (Fowler, 1952)	X				300–394	entire/70–500
*Zenionhololepis* (Goode & Bean, 1896)**	X	X	X	X	300–825	Cp, Tb/180–700

**Figure 3.**
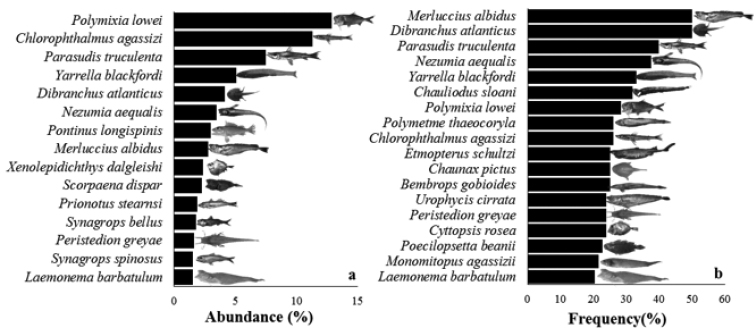
Abundance and frequency of the fish species: **a** Abundance (%) and **b** Frequency (%).

The most abundant species were *P.lowei* (1206 individuals), *P.truculenta*, *M.albidus*, *C.agassizi*, *D.atlanticus*, *N.aequalis*, *Y.blackfordi*, and *L.barbatulum*. Among these, *P.lowei* and *C.agassizi* are outstanding, with a relative abundance greater than 10%, and *D.atlanticus*, and *M.albidus* with a relative frequency of more than 50% (Fig. 3).

**Figure 4.**
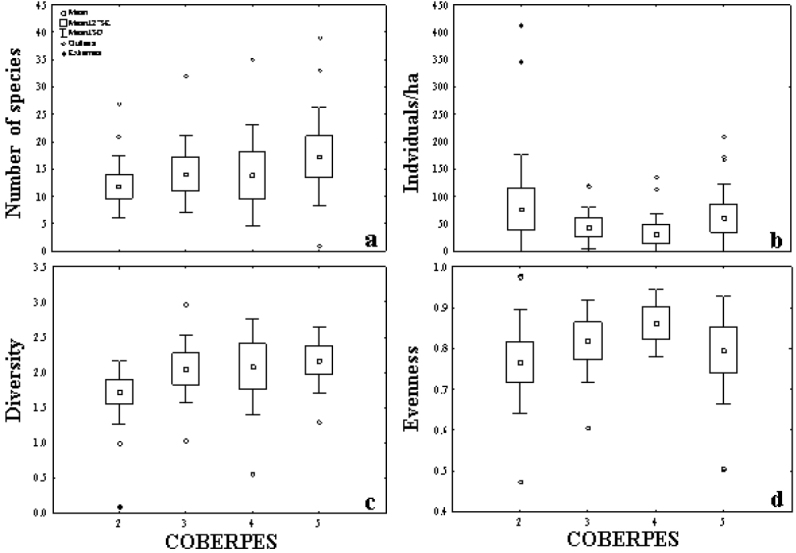
Community parameters for each cruise (COBERPES): **a** Species richness (number of species); **b** Abundance (individuals/ha); **c** Diversity (Shannon-Wiener); **d** Evenness (Pielou).

The lowest richness was found in the Yucatan slope area near the Caribbean Sea (COBERPES 2), with a total of 27 species and a mean of 11.81 ± 5.71 (SD) species per trawl, whereas, the highest one was registered in the Campeche Bay (COBERPES 5) with 39 species (17.26 ± 9.06 species per trawl), however, a high fish species richness (>30 species) was recorded at different sites throughout the GoM (Fig. 4a). The highest fish abundance was registered in the Yucatan continental slope, close to the Caribbean Sea (COERPES 2), with 412.46 individuals/ha recorded and a sample mean of 76.83 ± 19.18 individuals/ha (Fig. 4b). High fish diversity (Fig. 4c) and evenness (Fig. 4d) were recorded in several locations along the entire gulf, except in the area close to the Caribbean Sea (COBERPES 2).

Fifteen species extended their distribution into the continental slope of the southern GoM: *Eptatretuscaribbeaus* Fernholm, 1982; *Ventrifossamucocephalus* Marshall, 1973; *L.barbatulum*; *Brotulotaenianigra* Parr, 1933; *Lophiodesberoe* Caruso, 1981; *Hoplostethusmediterraneus* Cuvier, 1829; *Benthodesmussimonyi* (Steindachner, 1891); *Macroramphosusscolopax* (Linnaeus, 1758); *Bregmaceroscantori* Milliken & Houde, 1984; *Bregmaceroshoudei* Saksena & Richards, 1986; *Peristedionecuadorense* Teague, 196; *Peristedionthompsoni* Fowler, 1952; *Polyipnusasteroides* Schultz, 1938; *Neoscopelusmicrochir* Johnson, 1863, and *Neoscopelusmacrolepidotus* Matsubara, 1943 (Table 2).

**Figure 5.**
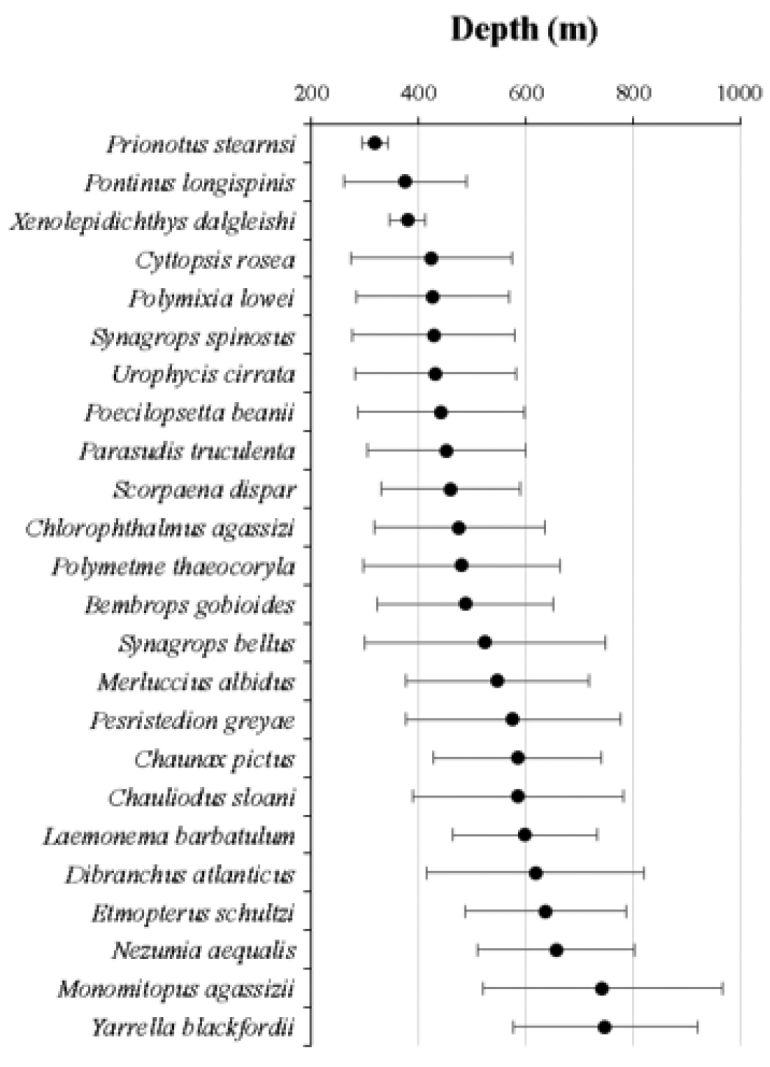
Depth of occurrence of the most abundant fish species: average depth ± standard deviation (SD).

Thirty seven species increased its depth range distribution (Table 2). Three of the most abundant species recorded an average depth lower than 400 m (Fig. 3): *Prionotusstearnsi* Jordan & Swain, 1885 (318 ± 24.57 m); *Xenolepidichthysdalgleishi* Gilchrist, 1922 (379 ± 33.05 m) and *Pontinuslongispinis* Goode & Bean, 1896 (376 ± 114.03 m). Many of the species showed a wide depth range distribution (400>800); however, only two of them presented the highest average depth: *Monomitopusagassizii* (Goode & Bean, 1896) and *Y.blackfordi* (743 ± 223.92 m and 749 ± 172.95 m, respectively) (Fig. 5).

## Discussion

The species accumulation curve suggests that we registered most of the fish species found on soft bottoms of the continental slope of the southern GoM. Nevertheless, since the species accumulation curve continued to increase, the inventory still appears to be inconclusive. This situation is congruent with the fact that the sampling effort in the GoM deep waters has been low, particularly in the south. We identified 177 species which represent 12% of the total fish species (1541) reported for all habitats in continental shelf and deep waters including demersal and pelagic fishes of the GoM(McEachran 2009). The only previous systematic study using a similar sampling gear was conducted in the northern GoM by Powell et al. (2003) who recorded 93 demersal fish species for the upper (315–785 m) and mid slope (686–1369 m).

Based on the fish list elaborated by McEachran (2009) we counted 335 benthic and demersal fishes for the continental slope of the GoM. This number is 30 % higher than the 235 summed from this paper and Powell et al. (2003) study. It must be noted that McEachran list includes fishes collected with other gears and also in other habitats, like hard bottoms. Nonetheless, three fish species can be added to McEachran list: *Kaliindica* Lloyd, 1909, following Powell et al. (2003) and two species found in this research *E.caribbeaus* and *B.nigra*. In this way, a total compilation of 338 species of benthic and demersal fishes can be listed for this ecosystem. Additionally, 15 species extended their distribution into the south of the GoM (Table 2). It must also be noted that 37 species extended their depth ranges, nine of them were recorded in deeper ranges and 28 species in shallower depths than previously reported in literature. Most of the species showed a wide distribution depth range which is consistent with the distribution pattern of deep water fishes.

The highest species richness recorded in the continental slope of the Campeche Bay (COBERPES 5), is probably influenced by the high freshwater discharge of the largest hydrological system in the southern GoM: Grijalva-Usumacinta during summer, which inputs 62% of the total freshwater to the mexican GoM (Day et al. 2004), similar to what Powell et al. (2003) found in the northern GoM, near the mouth of the Mississippi River. Likewise, the upwelling produced by cyclonic gyres in the Campeche Bank (De la Lanza-Espino and Gómez-Rojas 2004, Durán-Campos et al. 2017), could be playing an important ecological role. These factors together incorporate large concentrations of nutrients which may trigger local productivity, and subsequently the diversity of demersal fishes on the continental slope in this region. Fish richness and diversity difference between COBERPES 3 and COBERPES 5 could also be influenced by seasonal productivity variations due to current pattern change in the area.

Five species captured in this survey are of commercial importance in other parts of the world. *M.albidus* was one of the second most frequent species (50%) which accounted greatly to total biomass (72.296 kg) and presented relatively large sizes (total length = 103–555 mm). This species could have a fishing potential in the GoM, as it was an important fishing resource in the US Atlantic in the early 1990s, but its production decreased significantly over a 10-year period of exploitation (Traver et al. 2012). Other taxa of commercial interest in the Atlantic such as *H.mirabilis*, *H.dactylopterus* and particularly *A.carbo* (one individual), are important fishing resources in the central and northern regions of the eastern Atlantic Ocean (Bensch et al. 2009, Pajuelo et al. 2010). Another species registered in the present study was *L.gastrophysus*, which was a significant deep water fishing resource in Brazil from 2000 to 2007 (Álvarez et al. 2009). However, the fishing potential of these species in the GoM is still to be defined with further studies.

Compiling data of fish species of this study as well as from the literature (McEachran and Fechhelm 1998, Powell et al. 2003 and McEachran 2009), we found that the north and south parts of the GoM share 97% of the species recorded on soft bottoms of the continental slope of the whole gulf. On the other hand, more than 63% of the species (n = 44) recorded for the Caribbean Sea (n = 69) (Anderson et al. 1985, Saavedra-Díaz et al. 2004, Paramo et al. 2015) also occur in the GoM. McEachran (2009) pointed out that this fish similarity is influenced by fauna from the central Atlantic (the region between North Carolina and the Great and Lesser Antilles, including The Bahamas, Bermuda islands, and South America) due to the Loop Current effect that connects the Yucatan and Florida currents (Monreal-Gómez et al. 2004, NOAA 2016).

This result is consistent with the distribution of deep water fishes inhabiting large bathymetric areas due to more stable environmental conditions in these habitats (Clark et al. 2010). A similar distribution pattern has been recorded in several studies done in the world, for example in the Mediterranean Sea (Moranta et al.1998), in the Atlantic (Menezes et al. 2006, 2015; Magnussen 2002; Bergstad et al. 2012; Koslow 1993;Quattrini et al. 2015); in the Caribbean (Quattrini et al. 2017), and in the northern of the GoM (Powell et al. 2003).

Our results suggest that a high number of species dwelling on the continental slope are shared between the north and south of the GoM. We recorded an extension in distribution into the south of the GoM and also bathymetrically of several fish species. New records are highly likely to be increased if sampling effort continues both geographically and bathymetrically, since the species cumulative curve did not reach an asymptote. This research contributes to the knowledge of the deep water fish community of the GoM, never studied before in the southern region. However, information needs to be enhanced since deep water natural resources of the southern GoM could be subject to increasing human pressures in the near future.
